# Zinc oxide nanoparticles improve testicular steroidogenesis machinery dysfunction in benzo[α]pyrene-challenged rats

**DOI:** 10.1038/s41598-021-91226-y

**Published:** 2021-06-03

**Authors:** Niveen M. Daoud, Mohamed S. Aly, Omaima H. Ezzo, Naglaa A. Ali

**Affiliations:** 1 Veterinary Research Division, Animal Reproduction and A. I. Department, National Research Center, El-Buhouth Street, Dokki, Cairo, Egypt; 2 Medical Research Division, Hormones Department, National Research Center, El-Buhouth Street, Dokki, Cairo, Egypt

**Keywords:** Toxicology, Gene expression analysis, Small molecules

## Abstract

Zinc oxide nanoparticles (ZnO NPs) demonstrate potential positive effects on reproduction. However, their protective role against the reproductive toxicity pollutants has not yet been adequately studied at the molecular level. This study was designed to assess this objective using Benzo[α]pyrene B[a]P as reproductive toxic agent . Forty-eight mature male rats were randomly distributed into six groups: Group1 (negative control); Groups 2 and 3 (positive control I and II, wherein the animals were treated with 10 and 30 mg ZnO NPs/kg BW, respectively); Group 4 (B[a]P group; treated with 150 mg B[a]P/kg BW); and Groups 5 and 6 (subjected to B[a]P treatment co-administered with different concentrations of ZnO NPs). We investigated oxidative stress biomarkers; cholesterol side-chain cleavage enzyme (CYP11A1), steroidogenic acute regulatory protein (StAR), and 3β-hydroxysteroid dehydrogenase (3β-HSD) gene expression; testosterone levels; and histopathology of the liver, kidney, and testicles. The B[a]P-treated group showed significant deterioration in all reproductive parameters and displayed induced oxidative stress. ZnO NPs remarkably reduced oxidative stress, effectively upregulated the mRNA levels of CY11A1, StAR, and 3β-HSD, and improved the histological pictures in the examined organs. At their investigated doses and given their NPs properties, ZnO NPs demonstrated a marked ameliorative effect against the reproductive toxic effects of B[a]P. Further studies are needed to thoroughly investigate the molecular mechanisms of ZnO NPs.

## Introduction

Nanoparticles **(**NPs) are materials with at least one dimension ≤ 100 nm and have a large surface-to-volume ratio. This characteristic endows NPs with unique properties that enable them to interact more effectively with biological systems^1,2^. There is an increasing interest on the impact of NPs in the fields of human and veterinary sciences, particulary on whether could easily pass through the blood–brain and blood–testis barrier^3^. NPs have several applications such as their ability to inhibit the growth of bacteria^4^, fungs^5^and detect SARS-CoV-2 virus^6^, and treat cancer^7^. Zinc oxide (ZnO) NPs have become one of the most useful metal oxide NPs in various applications in the biological and animal sciences owing to their exceptional properties of biocompatibility, solubility, and low toxicity, as well as their being economical^8^. Their structural features allow ZnO NPs to mimic biomolecules that regulate cell cycle and cellular homeostasis. However, whether ZnO NPs are toxic^9^ or whether they play a stimulating role in the reproductive system is a great dispute among reproductive scientists. This big question has actually led to a conclusion stating that the effects of NPs depend on different factors, such as their size, concentration, morphology, synthesis process, and surface area, as well as on the tested cell type and organism. Small sizes, high concentrations, and high frequency of administration enhance the toxic effects of NPs^10^. Recently, there have been numerous studies that used ZnO NPs as a protective agent against the reproductive toxicity associated with chemotherapy drugs, with streptozotocin-induced diabetes, and with nicotine and lead oxide^11–15^.

Further studies are needed to elucidate the protective effects of Zn nanomaterials against environmental pollutants that induce reproductive dysfunction in males. Therefore, our study aimed to investigate the protective effect of different ZnO NP concentrations on male rats treated with benzo[a]pyrene (B[a]P).

B[a]P is a polycyclic aromatic hydrocarbon (PAH) and is the most widespread environmental contaminant produced from the incomplete burning of fossil fuels, from tobacco smoke, from diesel consumption, and from roasted foods^16^. Data conclusively showed that even low to moderate exposure to B[a]P exerts an endocrine-disrupting and deleterious effects on the male reproductive system and results in steroidogenic dysfunctions^17–19^. B[a]P increases the production of reactive oxygen species (ROS) and thus the oxidative stress, leading to increased lipid peroxidation and causing male infertility^20,21^.

In this study, we examined the expression levels of some important steroidogenic enzymes, namely, cholesterol side-chain cleavage enzyme (CYP11A1) (a Leydig cell-specific gene), steroidogenic acute regulatory protein (StAR), and 3β-hydroxysteroid dehydrogenase (3β-HSD), using the quantitative real-time PCR technique; we supported our results with our data on an array of oxidative stress biomarkers, on the serum testosterone levels, and on sperm count, and we validated our findings with histopathological examination. Our result may contribute new data on the protective effects of the investigated doses of ZnO NPs toward male fertility.

## Materials and methods

### Chemicals

**Figure 1:**
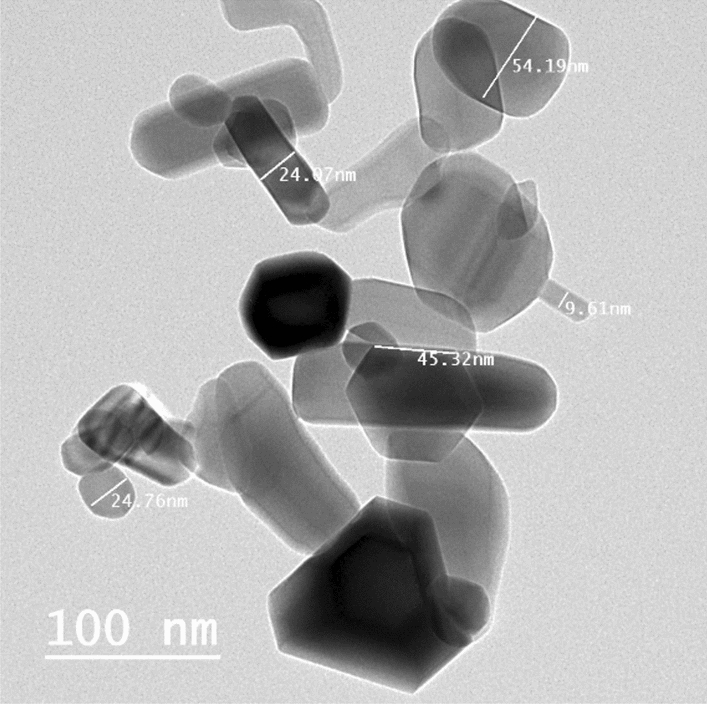
TEM of zinc oxide nanoparticles.

B[α]P and ZnO NPs (Product Code 544906) were purchased from Sigma-Aldrich Chemicals Co. (St. Louis, MO, USA). ZnO NPs have an average particle size of > 100 nm, a specific surface area of 10–25 m^2^/g, a formula weight of 81.39 g mol^−1^, and a quality level of 200. The actual surface area was determined to be 15.88 m^2^/g using the Brunauer–Emmett–Teller method^22^. They display high chemical stability, high electrochemical coupling coefficient, and high thermo-mechanical stability at ambient temperatures. The nanostructure of the ZnO NPs used were detected using a high-resolution transmission electron microscope (HR-TEM, JEM-1230, Japan) operated at 120 kV. The TEM images of the ZnO NPs are shown in Fig. 1. The TEM images revealed the hexagonal shape of ZnO, which explains the good characteristic of the ZnO NPs. In some places, spherical and tubular particles were found within and near the hexagonal particles. The average diameters of the NPs with different shapes ranged from 9 mm to 55 nm.

### Animal husbandry and experimental design

We purchased 48 adult male Wistar rats weighing 200–250 g from the animal unit of the National Research Center, Giza, Egypt. The rats were housed in stainless steel mesh cages in a naturally lit, ventilated room in the animal unit of the same institution. The lowest ambient temperature was adjusted to 30 °C ± 2 °C, and a 12 h light/12 h dark cycle was set; the rats were fed with a standard rat diet and provided with water ad libitum. The animals were allowed to acclimate to the new conditions for one week prior to the treatments.

The protocols and procedures employed in this experiment were approved by the Institutional Ethics Committee of the National Research Centre, Egypt, and the experiments were performed per the guidelines of the National Research Centre Ethical Committee for medical research and in compliance with the ARRIVE guidelines.

The rats were randomly allocated (**complete randomization**) into six treatment groups (n = 8 per treatment), as follows: Group 1 (**negative control**; **NC**) consisted of normal, healthy, untreated animals. Groups 2 and 3 were designated as **ZnONPs10 and ZnONPs30**, respectively; the animals in these groups served as positive control (PC) and were treated with 1 ml of 10 or 30 mg/kg BW/day ZnO NPs. Group 4 (**B[a]P group**) consisted of animals treated with B[a]P (98% HPLC purity) at 150 µg/kg BW/day. Groups 5 and 6 were designated as **B[a]P + ZnONPs10** and **B[a]P + ZnONPs30**, respectively; the animals were treated with B[a]P co-administered with different concentrations of ZnO NPs. All chemical preparations were given daily to the rats by oral gavage through oral cannula.

The ZnO NP dose of 10 mg/kg/BW applied in this experiment was based on the literature^13^, whereas the 30 mg/kg/BW dose was based on a published review that elucidated the impact of ZnO NPs on male (in)fertility^10^; the B[a]P dose was based on the work of Kang^23^. The selected doses were prepared by dissolving ZnO NPs in distilled water.

### Sampling

The animals were treated for 45 consecutive days. At the end of the experiment, the rats were subjected to sodium pentobarbital anesthesia for euthanization. We collected blood samples via sinus orbital puncture using un-heparinized pulled Pasteur pipettes. Subsequently, serum was collected after centrifugation and stored at − 20 °C until the assay for testosterone and oxidative stress biomarkers. Testis, liver, and kidney tissues were autopsied for histopathological examination, and epididymides were obtained for gene expression and sperm count determination.

### Determination of steroidogenesis-related genes using quantitative real-time PCR

#### RNA isolation and cDNA synthesis

The epididymides obtained from the rats were dissected and then frozen in liquid nitrogen. Total RNA was extracted after homogenization using the standard TRIzol® reagent extraction method (Invitrogen, USA). The RNA concentrations were determined at 260/280 nm using an ultraviolet spectrophotometer. Purified RNA obtained from a sample containing 500 ng of total RNA was immediately transcribed into single-stranded cDNA using a First Strand cDNA Synthesis Kit (MBI Fermentas, Germany) according to the manufacturer’s directions. Reverse transcription (RT) was performed at a total volume of 25 µl using 0.5 µl poly (dT)18 primer and 13 µl RNA.The reaction was run at 37 °C for 90 min and ended with a denaturation step at 70 °C for 15 min. The tubes containing the cDNA were stored at − 20 °C.

#### Quantitative real-time polymerase chain reaction (RT-PCR) using SYBR green I

The RT-PCR analyses for StAR, 3β-HSD, and cholesterol side-chain cleavage enzyme CYP450scc (CYP11A1 gene; Leydig cell-specific biomarker) were performed on an RT-PCR detection system (iQ5-Bio-Rad Laboratories, Cepheid, USA) using Syber green PCR master mix (TaKaRa Biotech Co., Ltd.). The expression levels of the gene mRNAs were normalized to a β-actin housekeeping gene (Actb). Our target gene and the Actb oligonucleotide sequence (Table 1) were based from published literatures^24,25^. The PCR reactions were performed in 25 μl reaction mixtures containing 12.5 μl 1 × SYBR, 0.5 μl forward primers, 0.5 μl reverse primer, 6.5 μl distilled water, and 5 μl cDNA template. The amplification cycle started with a preliminary denaturation step at 95 °C for 3 min followed by 35 cycles of denaturation at 95 °C for 15 s and then by an annealing step at 55 °C for 30 s. Finally, an extension step was performed at 72 °C for 30 s. Samples and controls were run in duplicate. The amplification was followed by a melt curve analysis to ensure that no primer–dimer amplification occurred. The gene expression levels were calculated using the formulae provided by Bio-Rad Laboratories, Inc.:*Ef* = 10 − 1/slope; Efficiency (%) = (Ef − 1) × 100^26^. We performed a relative quantification of the target to the reference by using the ΔC_T_ method provided that the E for our target genes and the reference primer (ACTB) are the same, that is, Ratio_(reference/target)_ = Ef^CT(reference)−CT(target)^.

**Table 1:** Primer sequences for RT-PCR.

Gene	Oligonucleotides sequence (5′–3′)	Accession Number
StAR	F: TCT CTA GTG TCT CCC ACT GCA TAG C R: TTA GCA TCC CCT GTT CG TAG CT	NM_011485.5
CYP11A1	F: ACAT GGC CAA GAT GGT ACA GTT G R: ACG AAG CAC CAG GTC ATT CAC	NM_019779
3β-HSD	F: ACAT GGC TCT GGG AGT TAT AAG GT R: TTA GTG ACT GGC AAG GCT TCT G	NM_008293
B-actin	AGA AGA TCT GGC ACC ACA CC TAC GAC CAG AGG CAT ACA GG	NM_007393.5

Abbreviations: F: forward primer; R: reverse primer. StAR: steroidogenic acute regulatory protein; CYP11A1: P450scc-cholesterol side-chain cleavage enzyme; 3β-HSD: 3β-hydroxysteroid dehydrogenase-1.

### Quantification of serum testosterone and oxidative stress biomarkers

The serum testosterone concentrations were determined using an enzyme-linked immunosorbent assay kit (XEMA Co., LTD, Moscow, Russia) according to the manufacturer’s instruction.

The level of serum malondialdehyde (MDA) was estimated colorimetrically using the thiobarbituric acid method according to the standard Ohkawa method^27^.

Reduced glutathione (GSH) activity was measured from the optical density of yellowish product of the reaction between GSH and DTNB (nitrobenzoic acid) according to the basic method^28^. We measured the serum MDA and GSH levels using the kits from Bio-diagnostic Co.

### Determination of sperm count

With the use of an established method^29^, the cauda epididymides were isolated, immediately immersed in normal physiological saline, gently shaken for 10 min, and then incubated for 2 min at 37 °C to allow the spermatozoa to leave the epididymal tubules. A solution consisting of 5 g sodium bicarbonate, 25 mg eosin, and 1 ml formalin (35%) dissolved in 100 ml distilled water was prepared and mixed with 1 ml supernatant fluid (1:100). An aliquot of this diluted sperm suspension (10 μl) was conveyed to each counting chamber of a hemocytometer and then counted under a light microscope at 200 × magnification.

### Histopathological examination

At the end of the experiment, testis, liver, and kidney samples were collected, immediately washed with normal saline, and then fixed in 10% formalin . Tissue samples were washed and then routinely processed, dehydrated in graded series of alcohol followed by clearance in xylol, and finally embedded in paraffin wax. Using a sledge microtome, we prepared 4–5 µm-thick specimens from the paraffin beeswax blocks and then stained them with hematoxylin and eosin^30^. We examined the histopathological changes under light microscope (Olympus CX 41, Japan). The abnormalities in the tissue sections were scored^31^ according to the Stevens’ scale, as follows: no damage (0) and mild (1), moderate (2), or severe pathological changes (3). Although the liver and kidney are not a concern of this study, they were used as reference in determining any cytotoxic effect of the ZnO NPs doses applied.

### Statistical analysis

We analyzed the data using IBM SPSS Statistics for Windows version 22.0 (New York, United States). Data are presented as mean ± standard deviation and statistically analyzed using one-way analysis of variance. Intergroup homogeneity was assessed using Duncan’s test. Statistical significance was set at *P* < 0.05. Pearson’s correlation linear regression was employed to test whether any correlation exists between the expression of steroidogenic enzymes and serum testosterone.

## Results

### MRNA gene expression levels of steroidogenesis-related genes as determined by RT-PCR

**Figure 2:**
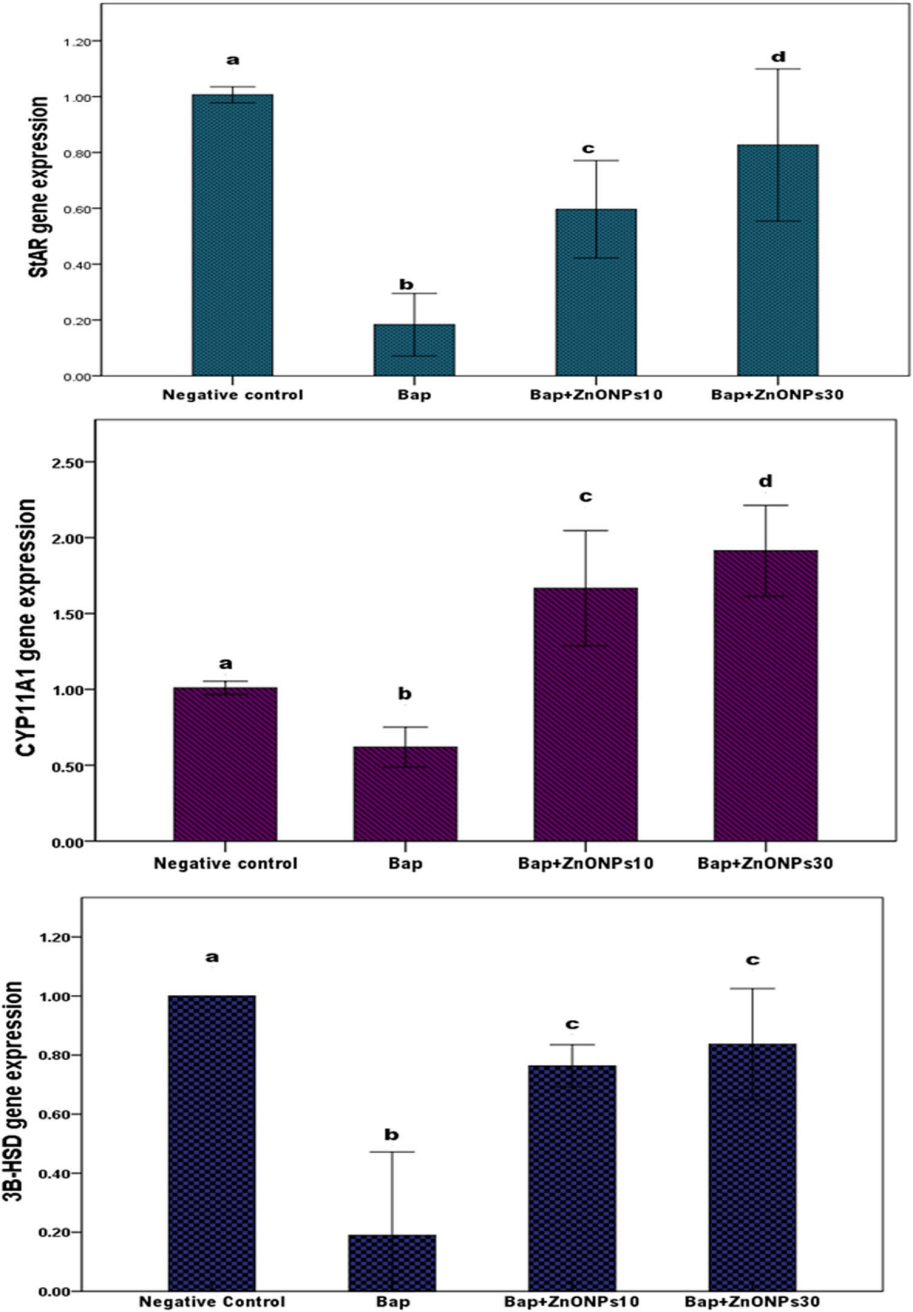
Effect of zinc oxide nanoparticles (ZnO NPs) in benzo[a]pyrene (B[a]P)-challenged rats. Results are presented as expression levels under the control of B actin. The B[a]P group showed a significant decrease in the expression levels of all steroidogenic enzymes compared with the negative control group. The co-administration of ZnO NPs with B[a]P significantly increased the expression levels of the steroidogenic enzymes compared with those in the B[a]P group, although the treatment could not restore the expression to the control levels. # Means with different superscripts (a, b, c, and d) indicate significant difference at P < 0.05. Abbreviations: StAR: Steroidogenic Acute Regulatory protein; CYP11A1: P450 Scc—cholesterol side-chain cleavage enzyme; 3β-HSD: 3β-Hydroxysteroid Dehydrogenase 1; B[a]P: Benzo[a]Pyrene; ZnO NPs: Zinc Oxide Nanoparticles.

The expression levels of the steroidogenesis-related enzymes (StAR, CY11A1, and 3 β–HSD) are shown in Table 2 and Fig. 2. No significant differences in gene expression levels were observed among the NC and PC groups (*P* > 0.05). The B[a]P group showed a significant decrease in gene expression levels, reaching as low as − 81.7% for StAR, − 61% for CYA11A1, and − 81% for 3β-HSD relative to those in the NC group (*P* > 0.001). Co-administration of ZnO NP supplementation with B[a]P resulted in a significant increase in the expression levels of the steroidogenesis-related enzymes relative to those in the B[a]P group. The gene expression levels of StAR significantly increased by 225.6% and 351%, those of CY11A1 increased by 167.3% and 207%, and those of 3 β-HSD by 301% and 340 in B[a]P + ZnONPs10 and B[a]P + ZnONPs30 groups, respectively (*P* > 0.001). The Pearson’s linear correlation between testosterone level and the expression levels of steroidogenic enzymes are highly significant (Table 2C). Although our results indicated an improvement in gene expression levels after exposure to B[a]P, they are still lower than those in the NC. These findings showed that ZnO NP supplementation promoted the expression of steroidogenic enzymes, which were inhibited by B[a]P; the ZnO NPs supplementation demonstrated a fairly good outcome, although it could not restore the gene expression to the control levels.

**Table 2:** Effect of zinc oxide nanoparticles (ZnO NPs) on the relative expression of steroidogenic enzymes in benzo [a] pyrene (B[a]P)-challenged male rats.

Groups	Genes		
	StAR	CY11A1	3β-HSD
**A. Statistical comparison among groups using ANOVA test**			
Negative Control (NC)	1.006 ± 0.0115	1.010 ± 0.0173	1.000 ± 0.000
ZnO NPs 10 (PC)	1.016 ± 0.015	1.006 ± 0.011	1.010 ± 0.010
ZnO NPs 30 (PC)	1.020 ± 0.010	1.003 ± 0.005	1.010 ± 0.010
B[a]P	0.183 ± 0.045*	0.621 ± 0.052*	0.190 ± 0.113*
B[a]P + ZnO NPs 10	0.596 ± 0.070*	1.660 ± 0.150*	0.763 ± 0.028*
B[a]P + ZnO NPs 30	0.826 ± 0.109*	1.910 ± 0.120*	0.836 ± 0.075*
**B. Statistical comparison among the B[a]P and co-administrated groups using ANOVA test**			
B[a]P	0.183 ± 0.045	0.621 ± 0.052	0.190 ± 0.113
B[a]P + ZnO NPs 10	0.596 ± 0.070*	1.660 ± 0.150*	0.763 ± 0.028*
B[a]P + ZnO NPs 30	0.826 ± 0.109*	1.910 ± 0.120*	0.836 ± 0.075*

	StAR	CY11A1	3β-HSD
**C. Pearson's correlation analysis between the expression levels of steroidogenic enzymes and testosterone**			
Pearson correlation coefficient with testosterone	*r* = 0.975	*r* = 0.392	*r* = 0.985
*P* value	0.000	0.022	0.000

Values are represented as mean ± standard deviation.

Values with superscript * within the same column means a significant difference from the NC group in table A and from B[a] P in table B at *P* < 0.05.

Abbreviations: StAR: Steroidogenic Acute Regulatory protein; CYP11A1: P450Scc cholesterol side-chain cleavage enzyme; 3β-HSD: 3β-Hydroxysteroid Dehydrogenase 1.

### Concentrations of antioxidant/oxidative stress indicators (MDA and reduced GSH)

**Figure 3:**
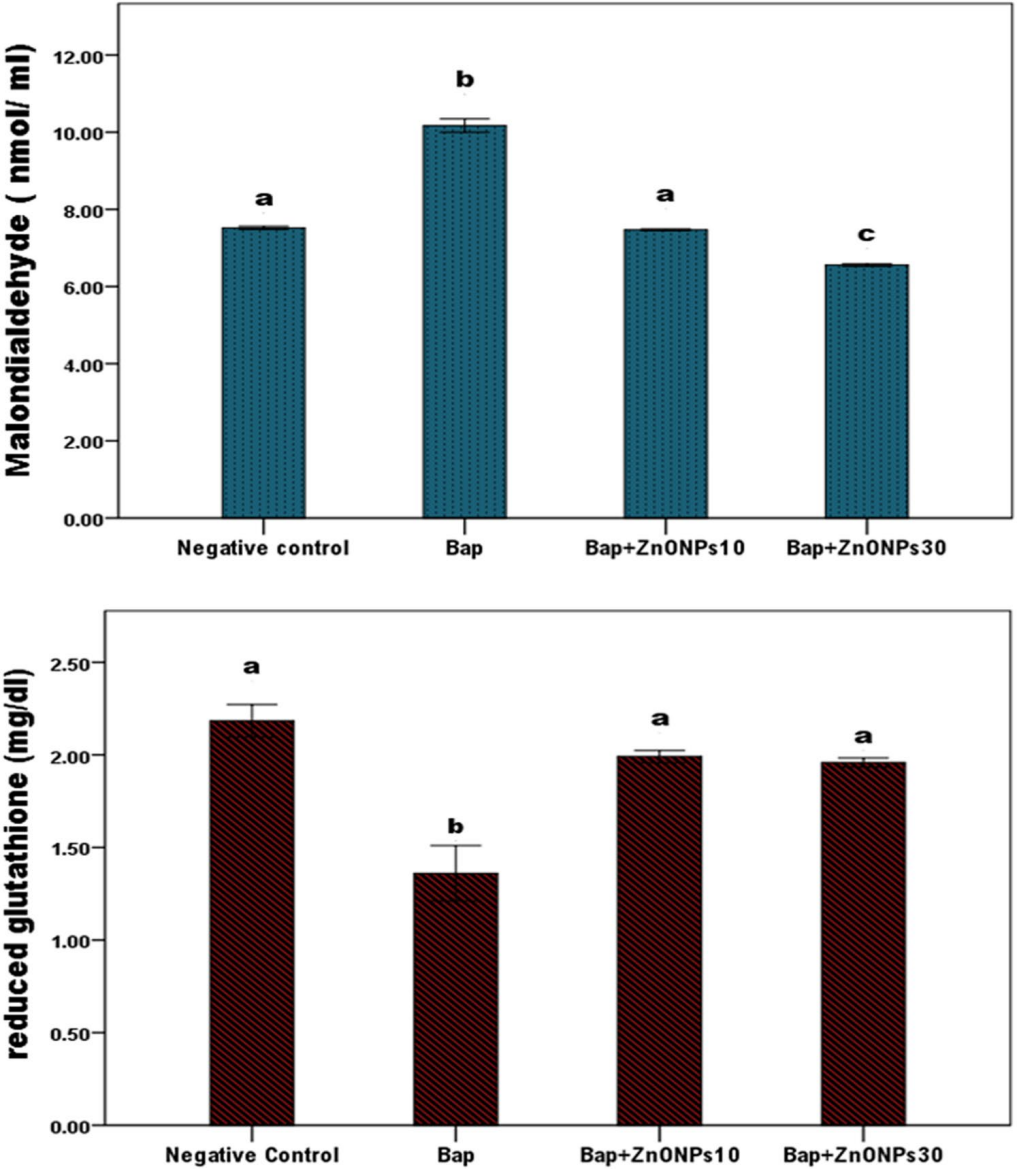
Antioxidant effect of zinc oxide nanoparticles (ZnO NPs) in benzo[a]pyrene (B[a]P)-challenged rats. The B[a]P group showed a significantly increased serum level of malondialdehyde (MDA) accompanied by a significant decrease in the level of reduced glutathione (GSH) compared with the negative control. The co-administration of B[a]P with ZnO NPs resulted in the significant decrease in MDA and an increase in the serum level of GSH, counteracting the effect of B[a]P and ultimately restoring the enzymes’ normal levels, especially GSH.Means with different superscripts (a, b, and c) indicate a significant difference at *P* < 0.

The quantification results for the oxidative stress markers, including MDA and GSH, are shown in Table 3 and Fig. 3. The levels in PCI and PCII significantly differed from those in the NC (*P* < 0.05). The B[a]P group recorded a significant increase in serum MDA by 35% and a significant decrease in GSH by − 37.6% compared with the NC (*P* < 0.05). In the co-administration of B[a]P and ZnO NPs (B[a]P + ZnONPs10 and B[a]P + ZnONPs30), an anti-B[a]P effect of ZnO NPs was observed, wherein the MDA level significantly decreased by − 26.5% and − 35.5%, respectively, and the GSH level increased by 46.3% and 43.3%, respectively, relative to those in the B[a]P group; moreover, the levels of the indicators return relatively to those in the NC, although the differences were not significant (*P* > 0.05). These findings showed that ZnO NPs supplementation exerted an antioxidant effect either at the level of negative control as seen in the comparison of the treatment group with the NC or as seen in the in the B[a]P group wherein ZnO NPs counteracted B[a]P and restored the oxidative stress biomarkers to the control levels.

**Table 3:** Effect of zinc oxide nanoparticles (ZnO NPs) on oxidative stress biomarkers in the serum of benzo [a] pyrene (B[a]P)-challenged male rats.

Groups	Parameters	
	MDA (nmol/ml)	GSH (mg/dl)
**A. Statistical comparison among groups using ANOVA test**		
Negative Control (NC)	7.52 ± 0.05	2.18 ± 0.10
ZnO NPs 10 (PC I)	6.10 ± 0.30*	2.77 ± 0.12*
ZnO NPs 30 (PC II)	6.24 ± 0.14*	2.80 ± 0.14*
B[a]P	10.17 ± 0.21*	1.36 ± 0.17*
B[a]P + ZnO NPs 10	7.47 ± 0.02	1.99 ± 0.03
B[a]P + ZnO NPs 30	6.55 ± 0.03*	1.95 ± 0.02
**B. Statistical comparison between the B[a]P and supplementation groups using ANOVA test**		
B[a]P	10.17 ± 0.21	1.36 ± 0.17
B[a]P + ZnO NPs 10	7.47 ± 0.02*	1.99 ± 0.03*
B[a]P + ZnO NPs 30	6.55 ± 0.03*	1.95 ± 0.02*

Values are presented as mean ± standard deviation.

Values with superscript * within the same column indicates a significant difference from the NC group in table A and from B[a]P in table B at *P* < 0.05.

Abbreviations: MDA: Malondialdehyde; GSH: reduced glutathione.

### Testosterone concentration and sperm count

**Figure 4:**
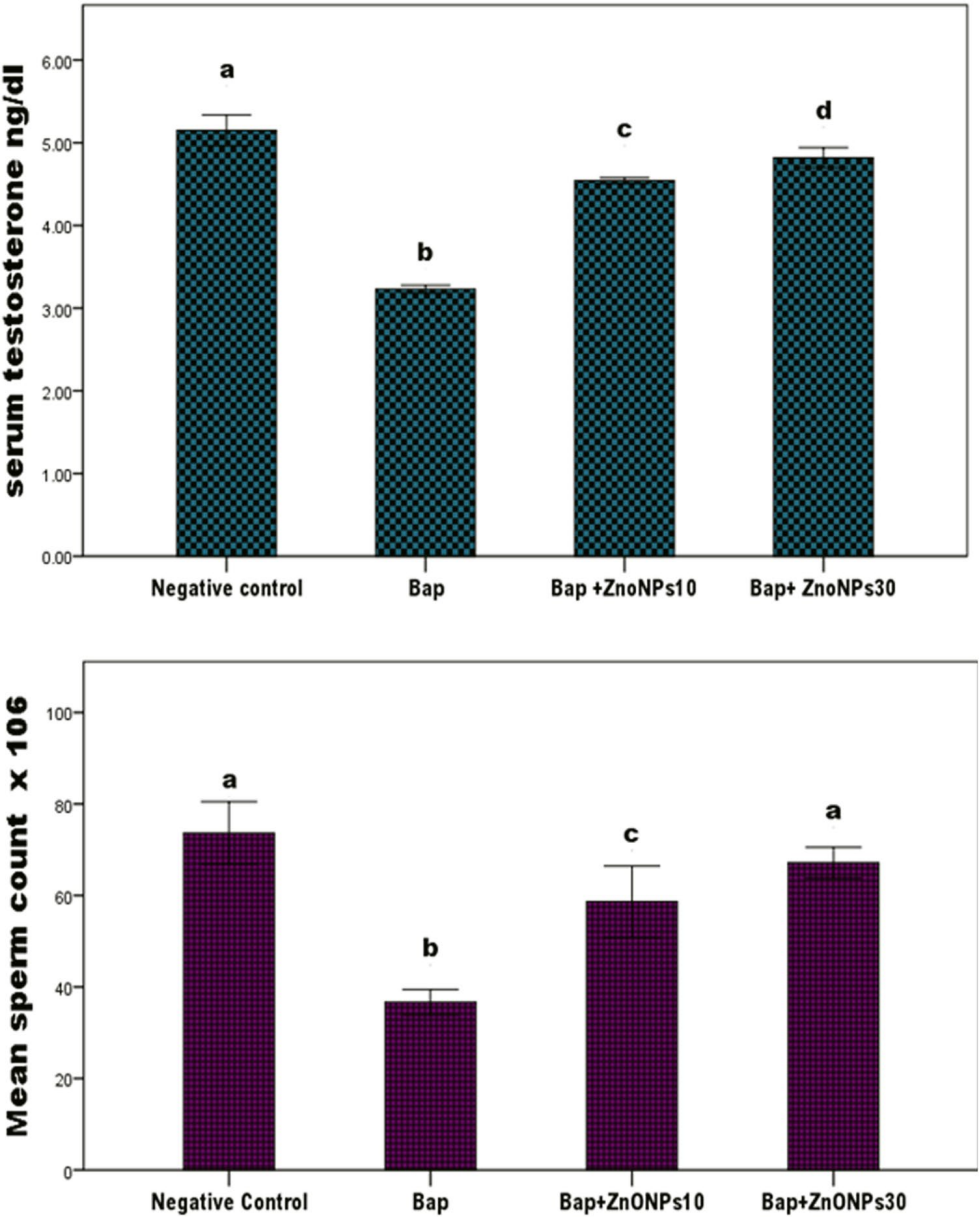
Effect of zinc oxide nanoparticles (ZnO NPs) on serum testosterone and sperm count in benzo[a]pyrene (B[a]P)-challenged male rats. The testosterone level and sperm count are significantly decreased in the B[a]P group relative to those in the negative control. The co-administration groups recorded a significant increase in testosterone concentration by nearly one-third and in sperm count by nearly half and two-fourth compared with the B[a]P group, demonstrating that ZnO NPs can protect sperm cells against the toxic effect of B[a]P. # Means with different superscripts (a, b, c, and d) between groups are significant at *P* < 0.05.

The statistical analysis results for testosterone level and sperm counts are shown in Table 4 and Fig. 4. Although no significant difference in testosterone levels were observed between the NC and PCI groups (*P* > 0.05), a significant increase in testosterone level was observed in the PCII group. The B[a]P group recorded a significant decrease in testosterone level (− 39.2%) relative to the NC. The co-administration groups (B[a]P + ZnONPs10 and B[a]P + ZnONPs30) reported a significant improvement in testosterone level by 40.5% and 48.9%, respectively, compared with the B[a]P group. However, the levels in the co-administration groups significantly differed from those in the NC.

**Table 4:** Effect of zinc oxide nanoparticles (ZnO NPs) on serum testosterone and sperm counts in benzo[a]pyrene (B[a]P)-challenged male rats.

Groups	Sperm count (× 10^6^/ml)	Testosterone (ng/dl)
**A. Statistical comparison among groups using ANOVA test**		
Negative control (NC)	73.66 ± 6.51	5.32 ± 0.18
ZnO NPs 10 (PCI)	68.83 ± 3.76	5.49 ± 0.12
ZnO NPs 30 (PCII)	73.83 ± 6.30	5.88 ± 0.11*
B[a]P	36.67 ± 2.65*	3.23 ± 0.05*
B[a]P + ZnO NPs 10	58.67 ± 7.44 *	4.54 ± 0.04*
B[a]P + ZnO NPs 30	67.17 ± 3.25*	4.81 ± 0.14*
**B. Statistical comparison among B[a]P and co-administration groups using ANOVA test**		
B[a]P	36.67 ± 2.65	3.23 ± 0.05
B[a]P + ZnO NPs 10	58.67 ± 7.44*	4.54 ± 0.04*
B[a]P + ZnO NPs 30	67.17 ± 3.25*	4.81 ± 0.14*

Values are presented as mean ± standard deviation.

Values with superscript * within the same column means a significant difference from the NC group in table A and from B[a]P  in table B  at *P* < 0.05.

Regarding the sperm count, there aren't significant differences among the NC and PC groups (*P* > 0.05). The sperm count in B[a]P group significantly decreased by − 50.2% (*P* < 0.001) relative to that in the NC. Meanwhile, the co-administration groups (B[a]P + ZnoNPs10 and B[a]P + ZnoNPs30) recorded a significant increase in sperm count (59.9% and 83.1%, respectively) compared with the B[a]P group (*P* < 0.001). However, the sperm counts remained significantly lower than that in the NC group. These results indicated that ZnO NPs supplementation stimulated the testosterone synthesis accompanied by an increase in sperm counts, which was inhibited by B[a]P; the supplementation demonstrated a fairly good outcome, although the sperm counts remained lower than the control levels.

### Histopathological findings

**Figure 5:**
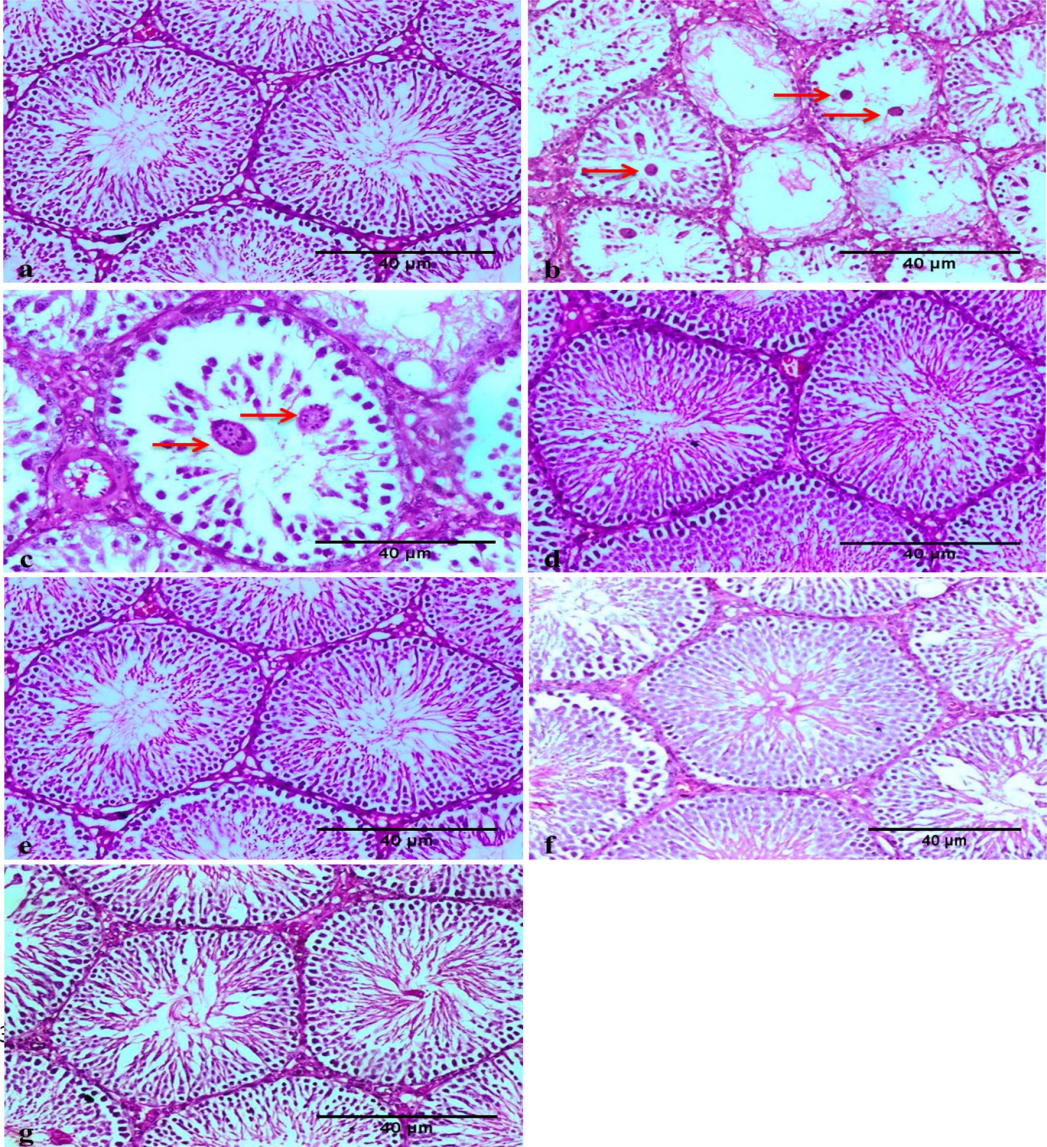
Photomicrograph showing the absence of histopathological changes in the testis of the control rat (**a**). The benzo[a]pyrene (B[a]P)-treated group shows severe desquamation, necrosis, and absence of spermatocytes and most Sertoli cells (Star) in the testis (**b**); these changes are associated with the formation of giant cells (symplast) (red arrows) (**c**) (H & E, 200 ×). The co-administration of zinc oxide nanoparticles (ZnO NPs) and B[a]P improved these histopathological alterations (**d** and **e**). Images showing a normal testis obtained from male rats treated with ZnO NPs (scale bar: 40 μm) (**f** and **g**).

Histopathological examination revealed a normal testicular histology in the control group (Fig. 5a). B[a]P administration altered the normal testicular structure by causing atrophy of the seminiferous tubules characterized by severe vacuolar degeneration and desquamation of spermatogonial cells lining the seminiferous tubules, reduced width of the adluminal compartment of the seminiferous tubules, lost integrity of cellular membranes, lack of spermatids and spermatozoa, and altered morphology of spermatogonia and spermatocytes with complete necrosis of spermatocytes and most of Sertoli cells (Fig. 5b), along with the presence of multinucleated spermatid giant cells (symplast) (Fig. 5c). Improvement in these histopathological alterations was noticed in all examined sections obtained from rats treated with ZnO NPs and B[a]P (Fig. 5d, e). The ZnO NPs treatment groups showed normal testicular histology (Fig. 5f, g).

**Figure 6:**
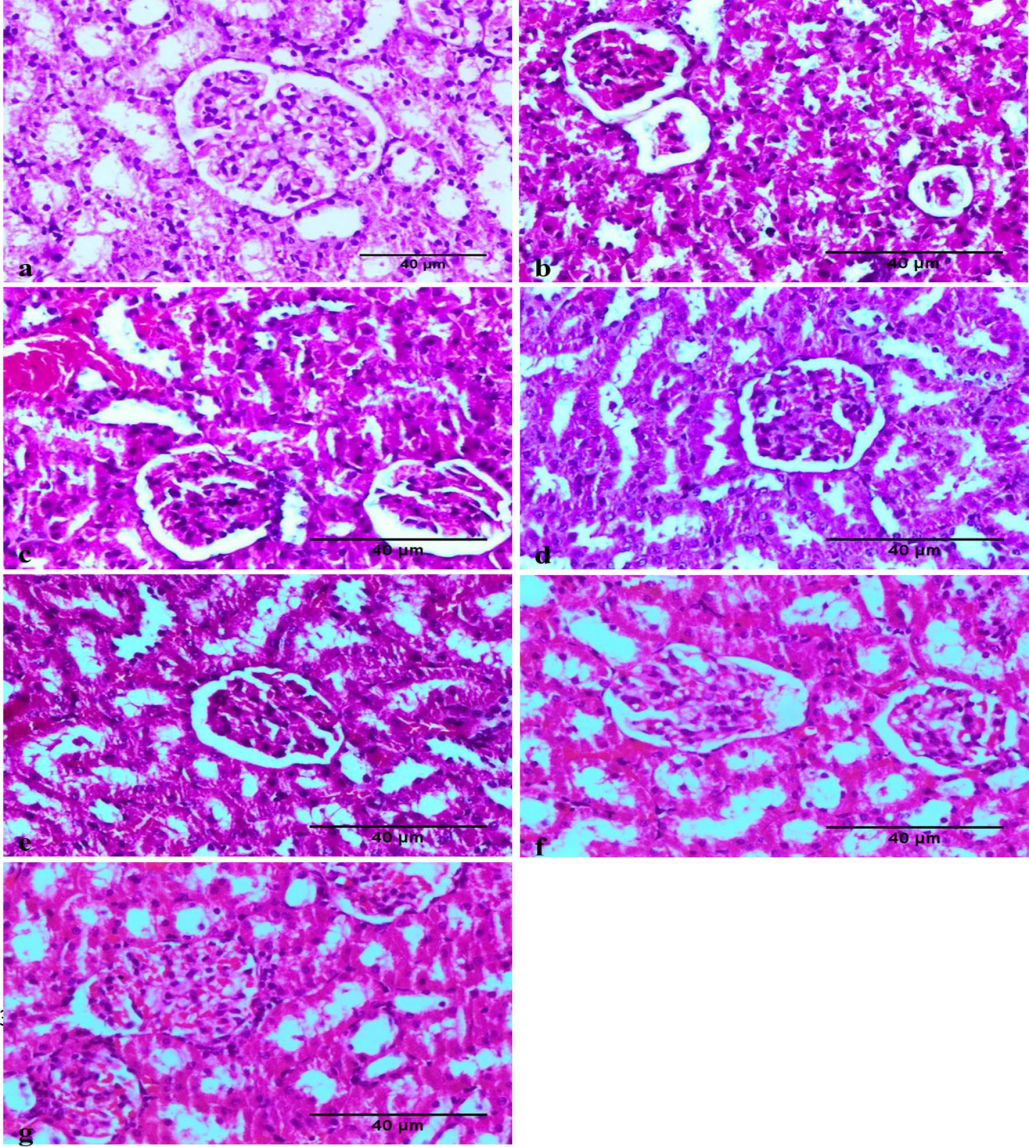
Photomicrograph showing the absence of histopathological changes in the kidney obtained from a rat in the negative control group (**a**). Section obtained from benzo[a]pyrene (B[a]P)-treated rats displaying severe necrosis and desquamation of renal tubular epithelial cells, atrophy, and degeneration of glomerular tuft with vacuolization in the Bowman’s space (**b**) and showing necrobiotic changes in the epithelial lining of the renal tubules, along with severe congestion (**c**). Images showing the apparent normal histologic features with mild individual cellular necrosis in groups exposed to zinc oxide nanoparticles (ZnO NPs) and B[a]P (**d** and **e**). Images showing the apparent normal histology with mild congestion and vacuolation of glomerular tuft in male rats treated with ZnO NPs (scale bar: 40 μm) (**f** and **g**).

Concerning the kidney, the sections in the control group showed a normal tissue architecture of the renal glomeruli (Fig. 6a). The B[a]P-treated kidney sections showed necrosis and desquamation of renal tubular epithelial cells, vacuolations and atrophy of glomerular tufts associated with hyalinization in the Bowman’s space (Fig. 6b), congestion of renal blood vessels, and degeneration of epithelial cells lining the renal tubules (Fig. 6c). The co-administration of ZnO NPs and B[a]P resulted in normal histologic features as well as in markedly prevented congestion in glomeruli and vessels and in other alterations characterized by mild individual cellular necrosis (Fig. 6d, e). Treatment with ZnO NPs did not cause histopathological changes, except mild congestion and vacuolation of the glomerular tuft (Fig. 6f, g).

**Figure 7:**
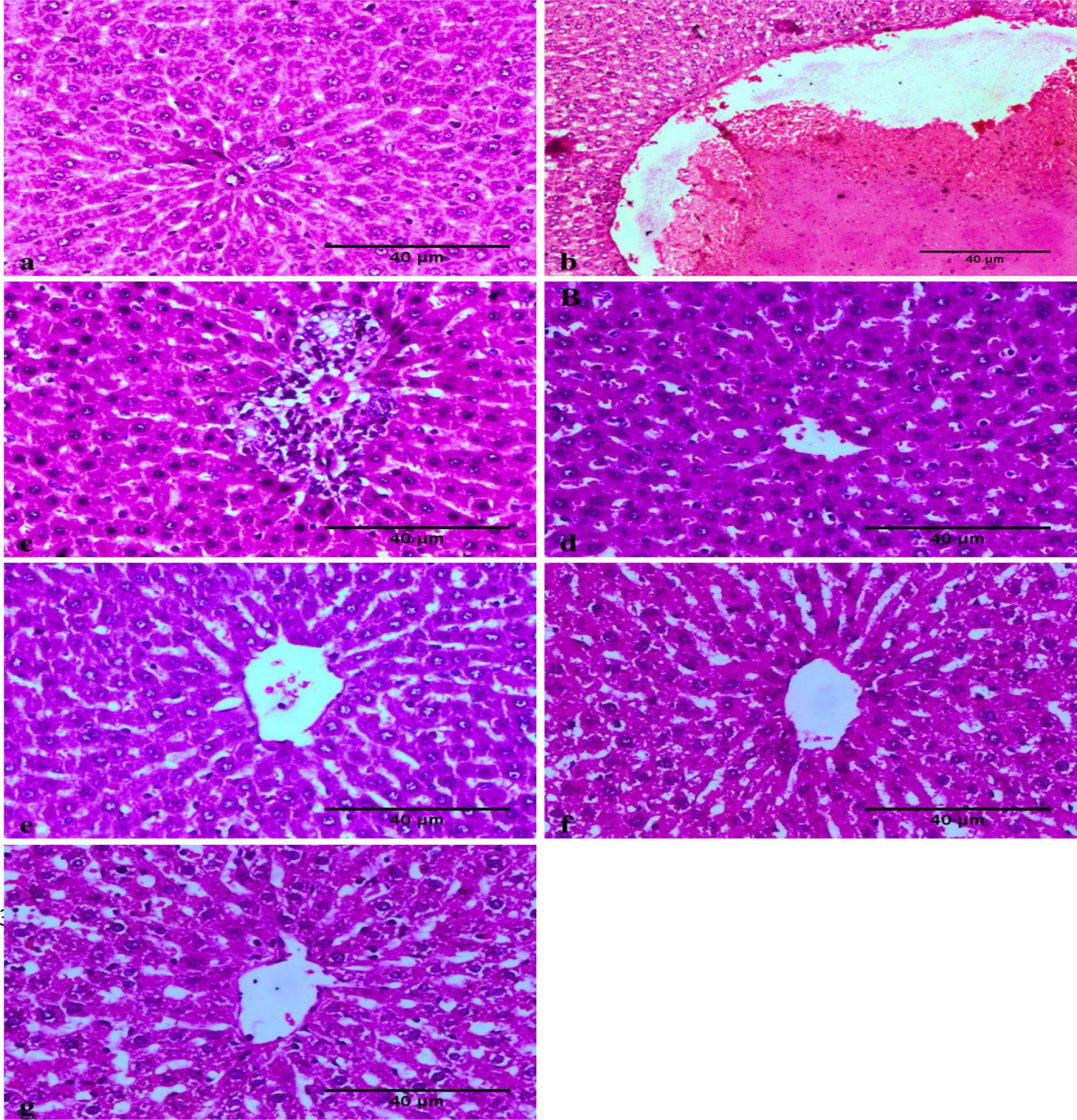
Photomicrograph showing the absence of histopathological changes in the of liver of negative control rats (**a**). In the benzo[a]pyrene (B[a]P)-treated group, disorganization of the hepatocytes and vascular dilation, congestion, and early non-occluding thrombus formation associated with hyalinization and thickening of blood vessel wall were observed (**b**). Degenerative changes in hepatocytes associated with leucocytic cell infiltration (**c**). A normal hepatic tissue was observed in a male rat exposed to zinc oxide nanoparticles (ZnO NPs) and B[a]P (**d** and **e**). A normal hepatic histology was observed in male rats treated with ZnO NPs (positive control groups) (scale bar: 40 μm) (**f** and **g**).

In the histopathological analysis of the liver, the control animals displayed no histopathologic changes (Fig. 7a). Meanwhile, sections from the B[a]P-treated rats showed an alteration in the cellular architectural pattern of the hepatic parenchyma associated with hepatic parenchyma cellular disorganization of the hepatocytes as well as vascular dilation and congestion. Some sections showed early non-occluding thrombus formation (Fig. 7b) and leucocytic cell infiltration of the portal area, along with the activation of Kupffer cells (Fig. 7c). Improvement in histopathological picture was noticed in the sections obtained from the rats treated with ZnO NPs and B[a]P (Fig. 7d, e). Furthermore, the examined sections of the animals treated only with ZnO NPs showed no histopathological alterations (Fig. 7f, g).

## Discussion

Available data conclusively proved that B[α]P reduces male fertility. ZnO is considered a multifunctional material due to its unique physical and chemical properties. It is known to be crucial for testosterone synthesis and spermatogenesis. However, studies on the effects of ZnO NPs on male fertility either in vitro or in vivo are still rare. Therefore, this study sought to evaluate the ameliorative effect of ZnO NPs supplementation on male fertility in B[α]P-exposed rat by determining its effect on the molecular, biochemical, and histological characteristics of tissues .

In our study, B[a]P induced severe oxidative stress, which significantly increased the MDA levels and decreased the GSH levels (*P* < 0.001). B[a]P is a PAH that undergoes intracellular biotransformation induced by cytochrome P450 (CYP) enzymes, leading to the production of ROS accompined by the reduction of antioxidant enzymes as reduced glutathione. Free radicals initiate lipid peroxidation through a chain reaction and thus increasing the levels of lipoperoxidation products, such as MAD^32^. This phenomenon correlates logically with oxidative stress^18,21^, consistent with our results.

ZnO NPs supplementation recorded an antioxidant stress as seen in the comparison of NC group or as seen in the B[a]P, wherein ZnO NPs counteracted B[a]P and restored oxidative stress biomarkers to the control levels. Zn is a core component of over 200 metalloenzymes, including antioxidant enzymes, and is a known protector of sulfhydryl groups; it is also thought to weaken lipid peroxidation, which characterizes the anti-oxidative stress properties of Zn^33,34^. As a dietary supplement (10 mg/kg/BW), ZnO NPs reduce the harmful effects of exposure to nicotine in rats by reducing oxidative stress, and it improves male fertility^13^. Moreover, lower doses of ZnO NPs (10 mg/kg/BW) demonstrate a protective effect toward the sperm of diabetic rats owing to the antioxidant properties of ZnO NPs, as they increase the activity and mRNA expression levels of SOD, CAT, and GSH and decrease the MDA levels in testicular tissue^14^. On the contrary, Hussein et al. found that ZnO NPs found that ZnO NPs demonstrated a reduced antioxidant capacity and increased oxidative stress, inducing severe reproductive toxicity in male rats. This result was attributed to the doses used by the authors, as they used 100 and 400 mg/kg/BW, which led to oxidative stress^35^. Again, we can go back to what has been stated in Pinho’s review, that is, the effects of ZnO NPs depend on their size, concentration, morphology, and surface area. At low concentrations, ZnO NPs act as antioxidant agents, whereas ROS generates and induces apoptosis at high concentrations^10^.

In our study, the testosterone level was indicated a highly significant correlation between the expression levels of steroidogenesis-related enzymes and testosterone levels as shown by the Pearson’s linear correlation. Cholesterol is translocated from the outside to the inside of mitochondrial membrane, and this process is mainly dependent on the pivotal role of the StAR protein. Cholesterol is oxidized by mitochondrial cytochrome P450 oxidase (P450scc; CYP11A1) and is converted into pregnenolone. Pregnenolone is oxidized by 3β-HSD leading to the formation of androstenedione, which in turn is reduced by other enzymes, resulting in the formation of testosterone^36^. In the B[a]P group, both the testosterone level and the expression levels of steroidogenic enzymes were significantly lower than those in the NC group (*P* < 0.001). Our results are consistent with previous observations showing that StAR, CY11A1, and 3β-HSD could be regulated by endogenous and exogenous agents, including environmental toxins, such as B[a]P, which affects LH-stimulated Leydig cells and the serum testosterone production^18,37,38^. In the same context, under oxidative stress conditions, ROS activates stress, leading to the decrease in the gene expression levels of StAR, CY11A1, and 3β-HSD^19^. This phenomenon implies a potentially strong negative correlation between oxidative stress and testicular steroidogenesis^39,40^.

The co-administration groups showed an increased testosterone level in parallel with the improved gene expression of steroidogenic enzymes compared with the B[a]P group. This finding is in agreement with that of Le et al., who reported that ZnO NPs induced the upregulation of genes and that the increase in gene expression was dependent on exposure time and concentration^41^. Recently, Bara and co-authors^42^ examined the direct effect of different concentrations of ZnO NPs in vitro on mouse testicular Leydig cells (TM3), and they found a significant amplification of the expression levels of steroidogenic enzymes (STAR and CYP11A1). Recently, Mohamed et al. reported that ZnO NPs supplementation at a dosage of 10 mg/kg/BW increased the testicular gene expression levels of StAR and cytochrome P450scc in parallel with the testosterone levels in nicotine-exposed rats^13^. By contrast, Tang et al.^43^ reported that ZnO NPs decreased the testosterone production through the downregulation of StAR. This difference is attributed to the dose used (50, 150, and 450 mg/kg). Although several literature have presented the protective effect of ZnO NPs against drugs or toxic substances, the molecular mechanism of ZnO NPs remains unknown, especially in vivo.

Sperm count in the B[a]P group significantly decreased (*P* < 0.001) compared with that in the NC . This result is predictable given that the testosterone levels were considerably decreased. Testosterone is the androgen in the testis that promotes spermatogenesis^44^. As an oxidative stress inducer, B[a]P damages the DNA in the sperm nucleus and increases apoptosis at a specific stage of the germinal cycle^45–47^. Our results were further supported by histopathological changes in the testis, specifically the deleterious effects of B[a]P toward the testis. The alteration in the architecture of the seminiferous tubules, the altered morphology of spermatogonia and spermatocytes, and the atrophy of seminiferous tubules showed that B[a]P interferes with the process of spermatogenesis^48,49^.

The co-administration of ZnO NPs improved the sperm count and the histopathological findings. These results agree with the other findings showing that the administration of ZnO NPs prevented testicular toxicity and sperm damage via an antioxidant mechanism against doxorubicin^12,50^ and nicotine in adult rats^13^.

Our histopathological findings, specifically the nephrotoxic adverse effects of B[a]P, including degeneration, atrophy of the glomerular tuft, necrosis of the epithelial lining, disorganization of the hepatic parenchyma, necrosis, and leucocytic cell infiltration, agree with the published findings^51–53^. Moreover , our findings may be interpreted in the light of a previous published study showing that B[a]P-induced increase in free radical and ROS production reinforces tissue damage and is considered the central causative factor for the pathological finding involving membrane lipid peroxidation and DNA mutations^54^.

Nano-ZnO is known for its antioxidant and anti-inflammatory properties. Its antioxidant activity mainly involves the neutralization and scavenging of free radicals^55–58^. This phenomenon is supported by its ability to protect cell membrane integrity by increasing the antioxidant enzyme levels and by decreasing the MDA and free radical levels^59^. Zn demonstrates anti-apoptotic properties that protect guard cells against different pro-apoptotic molecules^60^. Our histopathological findings are in line with these concepts, as all examined tissues displayed normal histology similar to that observed in the control group. This finding indicates that ZnO NPs are helpful for tissue regeneration that helps reverse the damage caused by B[a]P.

## Conclusion

Our findings demonstrated that ZnO NPs, at their investigated doses and given their properties, exerted an ameliorative effect against B[a]P by decreasing oxidative stress and by increasing the expression levels of steroidogenic enzymes, resulting in the repair of tissue abnormalities. This finding may offer a ray of hope in the fields of reproductive toxicology and nanomedicine. Further studies are needed to identify the mechanisms as to how ZnO NPs improve male fertility.

## Data Availability

The datasets generated and/or analyzed in this study are available from the corresponding author upon reasonable request.
